# Non-viral eNOS gene delivery and transfection with stents for the treatment of restenosis

**DOI:** 10.1186/1475-925X-9-56

**Published:** 2010-09-27

**Authors:** Luis A Brito, Saradha Chandrasekhar, Steven R Little, Mansoor M Amiji

**Affiliations:** 1 Department of Pharmaceutical Sciences, Northeastern University, Boston, MA 02115 USA; 2 Department of Chemical Engineering, University of Pittsburgh, Pittsburgh, PA 15261 USA

## Abstract

**Background:**

In this study, we have examined local non-viral gene delivery, transfection, and therapeutic efficacy of endothelial nitric oxide synthase (eNOS) encoding plasmid DNA administered using coated stents in a rabbit iliac artery restenosis model.

**Methods:**

Lipopolyplexes (LPPs) with eNOS expressing plasmid DNA were immobilized on stainless steel stents using poly(D,L-lactide-co-glycolide) (PLGA) and type B gelatin coatings. The gene-eluting stents were implanted bilaterally in the denuded iliac arteries and eNOS transfection and therapeutic efficacy were examined 14 days after implantation.

**Results:**

The results show that non-viral lipopolyplex-coated stents can efficiently tranfect eNOS locally in the arterial lumen assessed by PCR and ELISA. Human eNOS ELISA levels were significantly raised 24 hours after transfection compared to controls (125 pg eNOS compared to <50 pg for all controls including naked DNA). Local eNOS production suppressed smooth muscle cell proliferation and promoted re-endothelialization of the artery showing a significant reduction in restenosis of 1.75 neointima/media ratio for stents with lipoplexes encoding eNOS compared with 2.3 neointima/media ratio for stents with lipoplexes encosing an empty vector.

**Conclusions:**

These results support the hypothesis that a potent non-viral gene vector encoding for eNOS coated onto a stent can inhibit restenosis through inhibition of smooth muscle cell growth and promotion of a healthy endothelium.

## Introduction

Over 24 million individuals are diagnosed with a form of heart disease every year and cardiovascular disease and its complications continue to be associated with the highest rate of mortality in the United States [1]. Coronary atherosclerosis is a condition where plaque consisting of cholesterol, lipids, calcium, blood cells and other material traveling in the blood deposits on the arterial vessel wall [2]. As the plaque size increases it can become unstable and rupture, triggering a clotting cascade that can occlude the vessel, thus obstructing blood flow. The obstruction of blood flow in the coronary artery leads to myocardial ischemia and infarction, and can be fatal. Removal of the plaque deposit is commonly performed by catheter-based non-invasive techniques, such as percutaneous transluminal coronary angioplasty, atherectomy and stent implantation procedures. All of these procedures can lead to a complication called *restenosis*, where the vessel diameter decreases due to proliferation of smooth muscle cells or neointimal hyperplasia in the lumen of the artery [3,4]. The placement of metallic stents has become the treatment of choice due to the lower percentage of associated restenosis and the prevention of vessel recoil [4].

Nitric oxide (NO) is produced locally by a series of enzymes called nitric oxide synthases (NOS). In the vasculature, NO is a critical molecule in the vessel wall as it is responsible for a variety of different functions including inhibition of platelet adhesion and aggregation, inhibition of leukocyte chemotaxis, inhibition of smooth muscle cell growth and migration, vasodilatation, and re-endothelialization [5]. Due to the pleiotropic effect of NO in the arterial lumen, a number of NO releasing systems, including drugs and polymers, have been explored for clinical application. However, the short *in vivo *half-life of NO (~2-5 seconds) and the potential for cardiovascular side effects distal from the restenosis site have limited these agents. On the other hand, eNOS encoding plasmid DNA for gene therapy is an extremely attractive option for coronary restenosis due to the potential for sustained local NO production [6,7].

Gene-eluting stent studies employed a variety of methods including the incorporation of plasmid DNA within a polymer coating or including it within a viral vector that is associated with a polymer coating via an antibody [8,9]. Since the surface area of a stent is extremely small (< 1 cm^2^) any vector must be exceptionally potent. A recent publication by Sharif, *et al*. [8] has discussed local eNOS gene therapy using adenoviral vector embedded in stents for the treatment of restenosis. Levy's group has used an adenoviral vector coding for iNOS to inhibit restenosis [10]. However, viral vectors have a history of serious toxicity, and as such, their routine use in the clinic is questionable [11]. Non-viral vectors promise to have a high safety profile and can be designed to be efficacious in gene delivery and transfection by judicious selection of the material and construct design. Most of the published work on non-viral transfection has been performed with commercially-available cationic lipid transfection reagents such as Lipofectamine^® ^and Lipofectamine Plus^®^. The use of Lipofectamine^® ^or Lipofectamine^® ^Plus in gene delivery is not a clinically viable option due to the known toxicity and poor transfection efficiencies associated with these cationic lipid reagents [12].

In this study, we have utilized poly(beta-amino ester) (PbAE) pre-complexed plasmid DNA encapsulated in cationic liposomes to overcome the limitations of viral vectors and develop a safe and effective gene delivery system for eNOS encoding plasmid DNA in restenosis. The results from a previous study have shown that lipopolyplexes (LPPs) made with PbAE, plasmid DNA, and cationic lipids were effective in the delivery of reporter and therapeutic plasmid DNA in human aortic smooth muscle and endothelial cells and resulted in high transfection efficiency [13]. We have evaluated a PLGA/type B gelatin-coated stent with LPP encoding for eNOS in the New Zealand white rabbit iliac artery restenosis model for transgene expression and therapeutic efficacy.

## Materials and methods

### Stent Coatings and *In Vitro *Characterization

Plasmid DNA expressing endothelial nitric oxide synthase (eNOS-pVAX1 regulated by a CMV promoter, sample kindly supplied by Dr. Duncan Stewart, Division of Cardiology, University of Toronto, Toronto, Canada) [14] was cloned in *E. coli*, amplified, and purified at Elim Biopharmaceuticals (Hayward, CA). pVAX-1 was purchased from Invitrogen (Carlsbad, CA) and used as an empty vector control. LPPs were prepared according to an optimized method, published previously[16], using eNOS expressing plasmid complexed with PbAE and encapsulated in cationic phospholipid, 1,2-dioleoyl-3-trimethylammonium propane (chloride salt) (DOTAP). For fluorescence analysis of LPPs, rhodamine-labeled 1,2-dioleoyl-sn-glycero-3-phosphoethanolamine (rhodamine-PE) was included in the LPP formulation.

Relysis Mini Legend^® ^stainless steel stents were coated by depositing LPP-containing type B gelatin solution. The stents were coated with 40 μl of 638 μg/ml eNOS encoding plasmid DNA (eNOSpVAX1) in lipopolyplexes mixed with 80 mg/ml mannitol and 80 mg/ml type B gelatin and allowed to air-dry in a sterile environment. Subsequently, PLGA solution in acetone (200 mg/ml) was uniformly added on top of the dried gelatin coating. The amounts of PLGA were varied to obtain different coating thickness. Stents coated with rhodamine-PE LPPs were imaged with an Olympus BX61 (Center Valley, PA) epi-fluorescent microscope throughout the expansion process. *In vitro *LPP release studies from stents were performed by placing in phosphate buffered saline (pH 7.4) at 37°C. Periodically, samples of the release medium were removed and fluorescence was measured with a Biotek Synergy^® ^HT plate reader.

### *In Vivo *Implantation and Tissue Harvesting

The animal studies were performed according to an experimental protocol approved by the Institutional Animal Care and Use Committee at Northeastern University (Boston, MA, USA). These studies conform to the Guide for the Care and Use of Laboratory Animals published by the U.S. National Institutes of Health (NIH Publication No. 85-23, revised 1996). Male New Zealand White rabbits (2.5-3.5 kg body weight) were purchased from Millbrook Farms (Amherst, MA). The animals (N = 15) were housed singly and had access to food and water *ad libitum *prior to surgery. Drinking water was supplemented with 0.07 mg/ml of aspirin (Sigma Chemical Co., St. Louis, MO) for all animals to reduce sub-acute thrombosis. The animals were allowed to acclimate for at least 48 hours prior to surgical procedure.

Denudation of the iliac arterial endothelial layer and stent implantation was preformed according to the published procedure of Welt, *et al.*, [15]. Briefly, the animals were anesthetized with a combination of xylazine (5 mg/kg) and ketamine (35 mg/kg) administered by intramuscular injection into the anterior thigh. The animal was shaved and the surgical area was prepped with Betadyne (7.5% Povidone^®^-iodine) and isopropyl alcohol. At the time of stent implantation, an intravenous bolus of heparin at 100 U/kg dose was administered to prevent acute thrombosis. The femoral artery was exposed and a 1 cm section was isolated and ligated. An arteriotomy was performed in the femoral artery. Using a vein lifter (Becton-Dickinson, Franklin Lakes, NJ), the vessel was propped open and a three French Fogarty balloon catheter (Edwards Life Sciences, Irvine, CA) was inserted and fed up to the abdominal aorta, passing the iliac artery. The balloon was filled with 0.6 ml of air and passed through the iliac artery 3 times to ensure full endothelial denudation. Control bare metal stent, PLGA/gelatin-coated stent (gelatin only), naked eNOS plasmid in coated stent (eNOS gelatin), and empty vector-LPP stent (empty vector LPP) as well as the eNOS plasmid LPP-loaded stent (eNOS LPP), mounted on a balloon-mounted stent catheter, were fed in through the femoral artery and placed in the iliac artery on the same day as denudation (N = 5 vessels/group). All animals were selected randomly for stenting with 2 different stent formulations. The stent was expanded with 8 atmospheres of pressure and left in place for 30 seconds; the balloon was deflated and subsequently removed.

On day of sacrifice animals were sedated with ketamine/xylazine. Animals were sacrificed with 0.5 ml/kg of pentobarbital (Nembutal^®^) given intravenously. Immediately following sacrifice, the abdominal aorta was ligated and phosphate buffered saline was flushed through the bottom half of the animal for at least 10 minutes. Vessels were harvested, the stents were removed, the vessels were sectioned in half and immediately flash frozen in liquid nitrogen and stored at -80°C for transfection evaluation.

After 14 days animals in the efficacy study (N = 5 vessels/group) were sedated and injected with bromodeoxyuridine (BrdU, 50 mg/kg intravenous, Sigma, St. Louis, MO) 30 minutes prior to euthanizing. A subset of the animals was injected intravenously with Evans blue dye (Sigma) 30 minutes prior to sacrifice. Immediately following sacrifice, the abdominal aorta was ligated and phosphate buffered saline was flushed through the bottom half of the animal for at least 10 minutes. Vessels were harvested and stored overnight at 4°C in buffered formalin for transfection and therapeutic efficacy.

### eNOS Transgene Expression by qPCR and ELISA

Quantitative PCR was performed on half of the iliac artery tissue to assess the levels of mRNA transcript after transfection. Primers for the rabbit housekeeping gene hypoxanthinephophoribosyltransferase (HPRT) were designed as previously reported [16]. RNA was extracted by a single step phenol extraction method. cDNA was then made with the First Strand III synthesis kit (Invitrogen, Carlsbad, CA) according to the manufacturer's instructions. Real time PCR (ABI 7900HT; Applied Biosystems, Foster City, CA) was performed on the cDNA using a SYBR Green RT-PCR kit (Applied Biosystems). The results obtained from the RT-PCR reaction were then analyzed by comparative C_t _analysis for determination of relative human eNOS in the samples. PCR products were visualized on agarose gels.

The other half of each harvested vessel was homogenized in lysis buffer (T-Per, Pierce Rockford, IL) and centrifuged to remove non-soluble cellular components. A Quantikine^® ^sandwich ELISA (R&D Systems, Minneapolis, MN) for human eNOS was used to determine eNOS protein levels according to the manufacturer's directions in triplicate for each sample. Human eNOS levels were normalized to total protein levels assessed with a bicinchoninic acid (BCA) protein assay (Pierce).

### Measurement of Nitrite by Griess Reaction

Extracts generated for the ELISA were also used to determine NO products levels indirectly. Nitrite levels were measured using a commercial Griess reaction kit (Cayman Chemicals, Ann Arbor, MI). The assay was preformed according to the manufacturer's directions in triplicate for each sample. Nitrite concentrations in tissues were normalized to total protein levels in the extracts.

### Tissue Histology

Vessel sections were stained with Verhoffs elastin stain and counter stained with Van Giesons stain and were examined with Olympus BX61 (Center Valley, PA). Bright-field images were taken at 4× and 10×. Morphometric analysis of the vessels was performed at 4× using Image J software to measure the intimal and medial thickness.

### Tissue Immunohistochemistry

Arterial tissue immunohistochemistry was performed on 5 μm methacrylate embedded sections according to previously published reports [15,17]. Briefly, antibodies for Ram-11, BRD-U, CD-31 and alpha-actin (Dako, Carpinteria, CA) were incubated with the sections after heat induced antigen retrieval in citrate buffer after protein blocking. A secondary anti-mouse HRP-linked antibody was incubated with the samples after removal of endogenous peroxide activity. Slides were developed with a metal-enhanced diaminobenzidine substrate kit for visualization (Pierce Rockford, IL). Specimens were counterstained with H&E and bright-field images were taken with an Olympus BX61 (Center Valley, PA) at 20× and 40×. Area with positive staining was counted and the values were tabulated at 40× magnification. Total cell counts were calculated for each region. All values are reported as positive stained cells divided by the total number of cells.

### Data Analysis

All experiments were repeated at least three times and the results are reported as mean ± standard error of the mean. Students T-test was employed to assess statistical significance. Statistical analysis was performed in Graph Pad Prism^® ^(La Jolla, CA).

## Results

### LPP-Immobilized Stent Design

**Figure 1:**
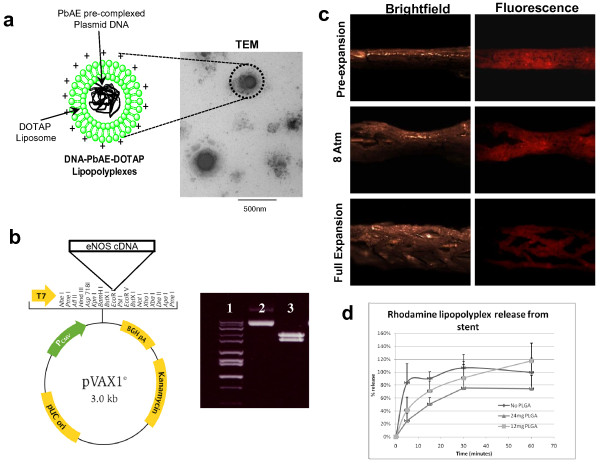
**Stent Coating with eNOS Plasmid DNA-Encapsulated Lipopolyplexes**. (A) Lipopolyplexes (LPPs) were prepared by plasmid DNA complexation with poly(beta-amino ester) followed by encapsulation in cationic liposomes. Transmission electron microscopy (TEM) analysis confirmed the size of approximately 200-400 nm and spherical shape. (B) Endothelial nitric oxide synthase (eNOS) expressing plasmid vector with 3.0 kB pVAX1 vector and cDNA encoding for human eNOS. Gel electrophoresis shows 1-10 kB ladder in lane 1, intact plasmid in lane 2, and the fragments after restriction enzyme digestion in lane 3. (C) Fabrication and expansion of LPP-immobilized type B gelatin-coated Relysis Mini Legend^® ^stainless steel coronary stents. LPP were formulated with rhodamine-labeled phospholipids for fluorescence microscopy. The stent expansion was initiated with up to 8 atmospheres of pressure over 5 seconds. (D) *In vitro *release profile of rhodamine-labeled lipopolyplexes from poly(D,L-lactide-co-glycolide) (PLGA)/type B gelatin-coated stents. Lipopolyplex release was examined from stents with no PLGA, with 12 mg of PLGA, and with 24 mg of PLGA coatings.

Figure 1a shows the schematic of the LPP formulation based on pre-complexation of eNOS encoding plasmid DNA with PbAE and encapsulation of the complex in DOTAP liposomes to form LPPs. Transmission electron microscopy (TEM) confirmed particle size analysis by light scattering indicating LPPs of approximately 200-400 nm in diameter [13]. The eNOS encoding plasmid, as shown in Figure 1b, was constructed using the 3.0 kB pVAX1 vector with full-length cDNA encoding for human eNOS. Agarose gel electrophoresis shows the intact plasmid and two bands after restriction enzyme digestion of the plasmid. When eNOS encoding plasmid DNA-containing LPP was immobilized on type B gelatin-coated stents, the resulting system, as shown in Figure 1c, had a uniform coating and could be expanded with up to 8 atmospheres of pressure over 5 seconds. The amount of DNA found on the stent was 15.85 ± 0.15 μg DNA/stent. This equates to a loading efficiency of 62.11% ± 0.60%. The *in vitro *LPP release data from the stents in Figure 1d showed that the release kinetics could be modulated by applying appropriate thickness of PLGA coating on the stent. For examples, when 24 mg of PLGA was applied per stent surface, 50% LPP release was prolonged for up to 20 minutes. Subsequent *in vivo *studies, therefore, were carried out with LPP-immobilized stents having type B gelatin and 24 mg of PLGA coating.

### Quantitative and Qualitative eNOS Transgene Expression

**Figure 2:**
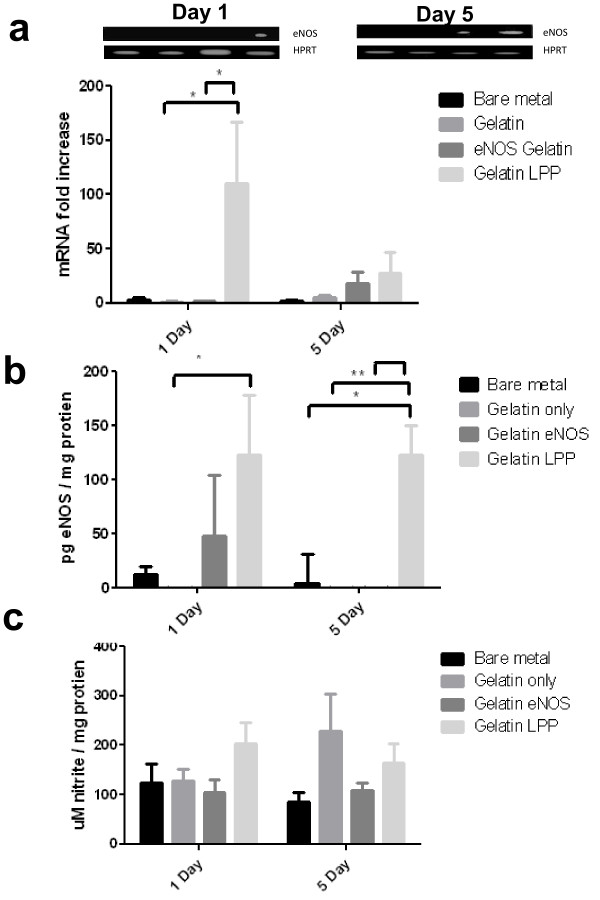
**Local Arterial eNOS Transgene Expression and NO Production**. (A) Quantitative PCR analysis of endothelial nitric oxide synthase (eNOS) mRNA expression in the rabbit iliac artery after 1 day and 5 days post-implantation of control and eNOS plasmid DNA-containing lipopolyplex (LPP) stents. (B) Transfected eNOS protein levels measured by ELISA were normalized to total protein content in the tissue samples. (C) Nitrite levels as measured by Griess reaction and normalized to total protein content in the tissue samples. The samples (n = 5 per group) represent bare-metal stents (bare metal), poly(D,L-lactide-co-glycolide) (PLGA)//type B gelatin-coated stent (gelatin only), naked eNOS plasmid embedded in PLGA/gelatin-coated stent (gelatin eNOS), eNOS LPP embedded in PLGA/gelatin-coated stent (gelatin LPP). * indicates p < 0.05, ** indicates p < 0.01, *** indicates p < 0.001 as assessed by ANOVA.

Quantitative PCR results from explanted rabbit arterial tissues are summarized in Figure 2a. At 1 day post-administration, there was a 100-to 150-fold increase in eNOS mRNA levels in the group administered with eNOS LPP-coated stents as compared to the control bare-metal stent. These groups also did not show any increase in mRNA levels. These results were also confirmed by the gel electrophoresis of the PCR products. Even after 5 days, there was a significant increase in mRNA transcript levels in the tissue from eNOS LPP-coated stent group. On both days, the levels of mRNA detected for the bare metal and gelatin groups were below the background of the assay. ELISA was used to measure eNOS protein levels after 1 and 5 days post-transfection from the explanted arterial tissue. Figure 2b summarizes eNOS protein expression levels. Negligible eNOS levels were found in the 1 day bare-metal and gelatin-coated stents groups and the 5 days bare-metal, gelatin only, and gelatin eNOS-coated stent groups likely due to low level cross-reactivity with rabbit eNOS. After 1 day, 50 pg eNOS/mg total protein was found in the gelatin eNOS-coated stent group. For both 1 day and 5 days post-transfection, eNOS LPP stents were found to have approximately 125 pg eNOS/mg total protein. One day after transfection there was significant levels of expressed eNOS in gelatin LPP stent groups (p < 0.05) as compared to stents coated with gelatin only. After 5 days, there was significant concentrations of eNOS after LPP mediated transfection when compared to bare-metal (p < 0.05), gelatin only (p < 0.01), and naked eNOS plasmid in gelatin (p < 0.001).

To assess whether the transfected eNOS was active in producing NO, a Griess reaction assay for nitrite levels was used. Due to the short half life of NO, the Griess reaction was used to measure long-lasting nitrate and nitrite metabolites. The results summarized in Figure 2c shows that 1 day after transfection, there was approximately 200 μM nitrite per mg of protein in the group given eNOS LPP-coated stents as compared to 110, 112 and 100 μM nitrite/mg of protein in the groups given the bare-metal, gelatin only and naked eNOS plasmid stents, respectively. After 5 days, the amount of nitrite in groups given bare-metal, gelatin only, naked eNOS plasmid, and eNOS LPP-coated stents was approximately 90, 210, 100 and 150 μM nitrite/mg of protein, respectively. Standard errors for the bare-metal, gelatin plasmid and gelatin LPP groups were similar to the 1 day time point. The gelatin only-coated group at day 5 exhibited a high variability with the standard error values ranging between 25 and 345 μM nitrite/mg of protein.

### Vessel Sectioning and Histology

**Figure 3:**
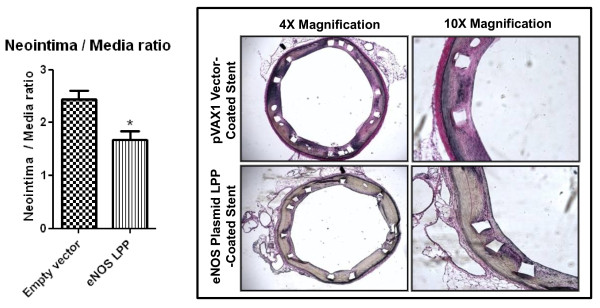
**Inhibition of Neointimal Hyperplasia**. Quantitative analysis from light microscopy images 14 days after implantation after polymethacrylate embedding of sections (A). Light microscopy images of rabbit iliac arterial tissue cross-sections after two weeks of control and endothelial nitric oxide synthase (eNOS)-expressing plasmid DNA-loaded stent implantation. Polymethacrylate-embedded 10 μm-thick arterial vessel sections were stained with Verhoffs elastin stain and counter stained with Van Giesons stain for measurement of neointimal-to-medial thickness ratios. Images were taken at 4× and 10× magnification (B).

To assess the therapeutic efficacy of eNOS LPP-mediated transfection, the neointimal-to-medial layer thickness ratio was calculated for each of the control and treatment groups (n = 5/group). Vessels were stained with Verhoffs elastin stain and counter stained with Van Giesons stain. Figure 3 shows representative images from each of the treatment groups at 4× and 10× magnification. Two weeks after the injury the neointimal-to-medial layer thickness ratio for each of the groups was 2.44 ± 0.37 and 1.68 ± 0.35 for the empty vector LPP, and eNOS LPP-coated groups, respectively. Vessel injury scores for the empty vector LPP, and eNOS LPP groups were 0.83 ± 0.14, 0.97 ± 0.04, respectively, and were not statistically different (p = 0.26 by ANOVA) (Table 1).

**Table 1:** Summary of Digital Morphometry and Immunohistochemistry Data.

Samples	Digital Morphometry	Immunohistochemistry		
	Neointimal-to-Medial Ratio (n = 5)	Percent Brd-U Positive (n = 3)	Percent RAM-11 Positive (n = 3)	Percent Alpha-Actin Positive (n = 3)

pVAX1 Empty Vector-Coated stent	2.44 ± 0.37	18.79 ± 5.54	16.32 ± 4.98	7.01 ± 1.40

eNOS Plasmid LPP-Coated Stent	1.68 ± 0.35*	9.67 ± 3.26*	16.32 ± 2.20	9.04 ± 5.48

Values are mean ± SEM, * P <0.05

### Tissue Immunohistochemistry

**Figure 4:**
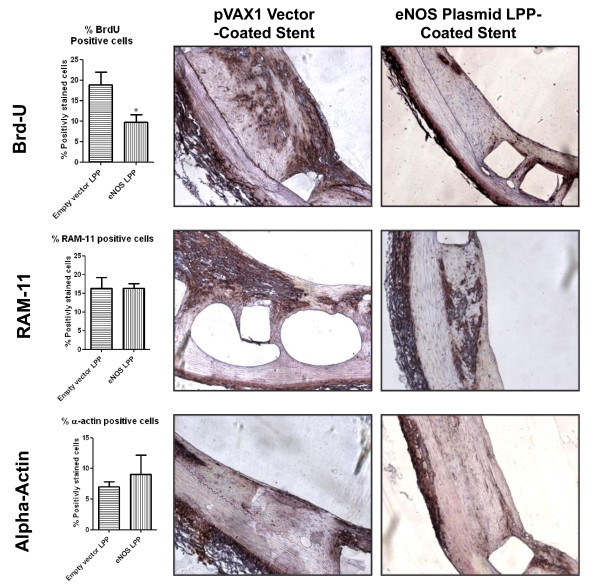
**Inhibition of Cellular Proliferation and Macrophage Activity**. Immunohistochemistry images at 20× magnification of rabbit iliac arterial tissue cross-sections after two weeks of empty vector and endothelial nitric oxide synthase (eNOS)-expressing plasmid DNA-loaded stent implantation. Tissue sections were treated with primary antibodies against Brd-U, RAM-11, and alpha-actin, followed by horse-radish peroxidase-conjugated secondary antibody, and stained brown with diaminobenzidine.

Tissue immunohistochemistry for cell proliferation, macrophage infiltration, and smooth muscle cell growth was performed on 5 μm-thick cross-sections using Brd-U, RAM-11, and alpha-actin specific antibody-based staining, respectively. Following two weeks of control and eNOS-LPP stent implantation, the vessels were sectioned for immunohistochemical analysis. The 20× magnification images in Figure 4 represent cells positively stained for Brd-U, RAM-11, and alpha-actin, graphs indicate % positively-stained cells. Positively-stained cells were counted and the percentage was calculated relatively to the total number of cells per given microscopic field of view, the results are summarized in Table 1. In the Brd-U stained samples, the pVAX1 empty vector-coated stent each showed higher percentage of diaminobenzidine staining within the intima indicating continued cell growth two weeks after implantation compared to eNOS LPP.

The presence of infiltrating macrophages within the intima was evaluated with RAM-11 antibody staining. The amount of stained cells were similar for both groups. Additionally, to assess smooth muscle cell proliferation an antibody for alpha-actin was used. Smooth muscle cells were only counted within the neointimal layer. The pVAX1 empty vector-coated and the eNOS LPP-coated groups showed a similar number of positive cells.

### Assessment of Re-endothelialization

**Figure 5:**
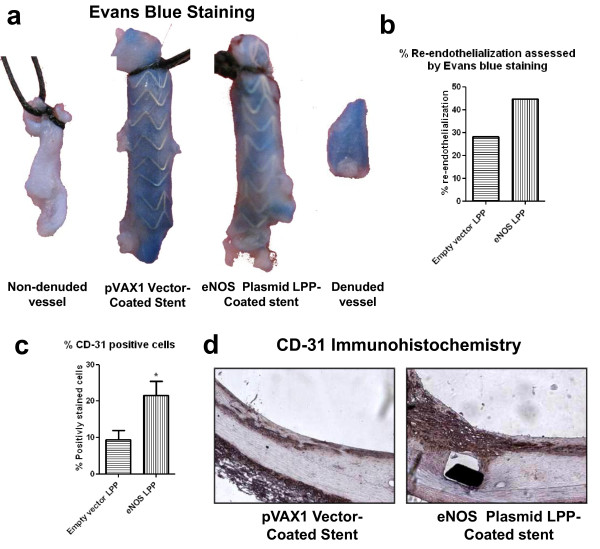
**Re-endothelization of the Iliac Arterial Lumen**. (A) Control and denuded rabbit iliac artery stained with Evans blue dye after two weeks of empty vector and endothelial nitric oxide synthase (eNOS)-expressing plasmid DNA-loaded stent implantation. (B) Quantitative analysis of percent re-endothelization based on the analysis of Evans blue staining of treated tissue relative to non-denuded control tissue normalized to surface area. (C) Histogram of quanitative analysis from CD-31 immuno-histochemistry. (D) CD-31 tissue immune-histochemistry at 20× magnification of the iliac artery cross-sections.

Luminal re-endothelialization was assessed two weeks after injury and stent implantation with Evans blue dye. While control (non-denuded) artery was completely white, areas within the denuded vessel exhibiting blue color indicate a lack of endothelium. Figure 5a shows optical images of the control and the experimental groups after Evans blue staining (N = 1). Quantitative analysis of Evans blue stained areas showed that the bare-metal stent had small areas of white tissue: analysis showed that this white area only represents 8% of the total area (Figure 5b). Upon examination of the empty PVAX1 vector LPP group, 28% re-endothelialization was observed. On the other hand, eNOS LPP group had vessels that showed the largest portion of white tissue; 45% of the vessel was determined to be re-endothelialized based on Evans blue staining.

CD-31 immunohistochemistry was used to as a marker for endothelial cells in the arterial lumen. Figure 5d shows CD-31 positive cells in the control and treated arterial sections (20X). Tissue from stents treated with the empty pVAX1 vector showed a small percent of brown diaminobenzidine stained cells, whereas the eNOS LPP-coated stents display a higher percentage of positively stained cells (Figure 5c).

## Discussion

Current clinically approved therapies for the inhibition of restenosis all use cytostatic or cytotoxic drugs that inhibit smooth muscle cell growth in the lumen [18]. These DES are very effective in inhibiting cell proliferation, but do not promote re-endothelialization of the vessel, which is a key step in the long-term treatment of restenosis [19,20]. NO is known to play a role in both inhibition of smooth muscle cell growth and re-endothelialization. A number of studies have investigated the effect of NO release from a polymer matrix for the treatment of restenosis [21,22]. Additionally, stent-based gene delivery studies have also been performed using viral vectors for transfection of eNOS and iNOS [6,8,10,16,23-33]. However, viral vectors are prone to significant toxicity.

There are a number of challenges for *in vivo *arterial gene delivery with a non-viral gene delivery system; the small surface area available at the lesion site, the residence time of the delivery system, cellular uptake of the system, and intracellular stability of the nucleic acid construct, efficient transcription, and efficient translation of the encoded protein. We have previously examined cellular uptake and transfection potential of LPP-based non-viral gene delivery system in human aortic smooth muscle and endothelial cells (18). After evaluating various formulation compositions, an optimized LPP system was formed by combining PbAE, plasmid DNA, and DOTAP in a 1:1:5 weight ratios. Additional studies were performed with immobilized LPPs on stainless steel meshes and stents to examine cellular uptake and preliminary *in vivo *transfection with reporter plasmid expressing green fluorescent protein [34].

In this study, eNOS expressing plasmid DNA in LPP formulations was immobilized on Relysis Mini Legend^® ^stainless steel stents using type B gelatin coating. The PLGA coating on the stent was used to prevent any premature dissolution of the gelatin layer during implantation. *In-*vitro release of LPPs indicated a very fast release from the stent surface, though *in vivo *release may be different particularly when the stent expands. We designed the system this way as to limit the resonance time of the lipoplexes. We have previously shown that these lipopolyplexes associate and enter cells very rapidly [13,34]. Once in the cell the vector would transfect the cells for an extended period of time. The coated stents were implanted bilaterally in the denuded rabbit iliac artery restenosis model. On day-1 and day-5 post-implantation, efficient eNOS transgene expression was observed at the mRNA and protein levels with the use of LPP-coated stents. The variability in mRNA transcript levels have also been observed by other investigators using nucleic acid constructs encoding for eNOS and iNOS delivered with viral vectors [32]. The naked eNOS plasmid entrapped in gelatin coating also showed high levels of mRNA and proteins. Other groups have shown that naked plasmids are able to transfect cells when embedded within a gelatin matrix, albeit at much higher concentrations [35-37]. Additionally, the Griess reaction results showed an increase in NO production for up to 5 days post-implantation of the coated stents. Gelatin-coated stents also showed relatively high levels of nitrite products. Since gelatin is well known to attract endothelial progenitor cells, the increased NO levels could be due to an increase in the accumulation of these cells [38]. Variability observed in the Griess data may be due to NO being generated by iNOS from inflammatory cells. Overall, the results from the qPCR, ELISA, and Griess reaction indicate that the eNOS plasmid formulated in LPPs and coated on the stent surface was efficient in entering and transfecting within the arterial lumen to produce sufficient concentrations of eNOS, which could lead to an increase in NO levels for up to 5 days after implantation. Presence of NO generated by inflammatory cells, also indicates that these immune cells are likely producing pro-inflamatory cytokines negating any positive effect of the NO.

The efficacy of eNOS gene therapy using LPP-coated stents was determined by calculating the neointimal-to-medial thickness ratio. The eNOS LPP group showed a significant decrease in this ratio, indicating a lower amount of intimal growth compared to the empty vector LPP. Fishbein *et al.*, looked at the effect of a iNOS transgene expression viral vector coated on a stent and saw a significant reduction in the neointima-to-media ratio of the experimental stent compared to a bare-metal stent only [39]. A recent study by Sharif *et al.*, examined eNOS transfection with a viral vector coated onto a stent [8]. The results showed a non-significant decrease in percent stenosis when a reporter vector or coating was compared to eNOS encoding viral vector in a normal rabbit model. The small differences in efficacy between groups seen in this study are common with normal rabbit model since their vessels do not show the same degree of restenosis upon denudation as the hypercholesterolemic model.

Cellular proliferation assessed by Brd-U immunostaining showed a decrease in proliferating cells two weeks after injury in vessels receiving eNOS LPP stents. The other groups exhibited similar amounts of cellular proliferation, indicating continuing cell proliferation in the empty vector LPP stent group. RAM-11 and alpha-actin positive cells in the empty vector LPP and eNOS LPP groups were similar for both of these treatment groups. CD-31 staining shows a high density of endothelial cells in the eNOS LPP group. This may be due to the presence of the gelatin acting as a seeding matrix for endothelial cells in the presence of the NO being produced.

Lastly, we have examined re-endothelization potential upon transfection with eNOS encoding plasmid DNA using the LPP formulation. The stents coated with gelatin only or eNOS LPPs show a higher percent of re-endothelialization as compared to the pVAX1 empty vector group. Another study employing viral vectors encoding for eNOS showed 80% re-endothelialization after 14 days [8]. Additionally, Cooney *et al.*, compared local delivery of a viral vector encoding for eNOS and iNOS and saw 90% and 75% re-endothelialization, respectively, 2 weeks after the injury [32]. The immunohistochemistry data for CD-31 further supports this, indicating that eNOS transfection leads to an increase in re-endothelialization.

We have successfully developed a non-viral plasmid DNA vector encoding for eNOS that can be coated onto a stainless steel stent and delivered locally for efficient transfection at the arterial luminal surface and a statistical decrease in lumen size. These results confirm what has been observed with catheter delivered viral and non-viral vectors in addition to stent based therapies [8,23,25,27,28,30,32,33,40]. Additional pre-clinical transfection and efficacy studies in a hypercholesterolemic rabbit or porcine model are warranted on this work since it is important to test this system in diseased vessels as transfection efficiency and efficacy of the gene therapy system may change in atherosclerotic vessels.

## Competing interests

The authors declare that they have no competing interests.

## Authors' contributions

LB performed the laboratory work, animal surgery, designed experiments, and drafted the manuscript. SC assisted with surgery and performed release assays. SL provided the polymer samples and consulted on the project. MA is the Principal Investigator who conceived the study, participated in its design and coordination, and helped to draft the manuscript. All the authors read and approved the final manuscript.
